# Seasonal Effect on the Biological Activities of* Litsea glaucescens* Kunth Extracts

**DOI:** 10.1155/2018/2738489

**Published:** 2018-02-20

**Authors:** Julio César López-Romero, Humberto González-Ríos, Aida Peña-Ramos, Carlos Velazquez, Moises Navarro, Ramón Robles-Zepeda, Evelin Martínez-Benavidez, Inocencio Higuera-Ciapara, Claudia Virués, José Luis Olivares, Zaira Domínguez, Javier Hernández

**Affiliations:** ^1^Centro de Investigación en Alimentación y Desarrollo (CIAD), 83000 Hermosillo, SON, Mexico; ^2^Departamento de Ciencias Químico Biológicas, Universidad de Sonora, Rosales y Luis Encinas, 83000 Hermosillo, SON, Mexico; ^3^Centro de Investigación y Asistencia en Tecnología y Diseño del Estado de Jalisco, A.C., CONSORCIO-ADESUR, Av. Normalistas 800, Colinas de la Normal, 44270 Guadalajara, JAL, Mexico; ^4^Centro de Investigación y Asistencia en Tecnología y Diseño del Estado de Jalisco, A.C., Clúster Científico y Tecnológico Biomimic®, Carretera Antigua a Coatepec No. 351, Colonia El Haya, 91070 Xalapa, VER, Mexico; ^5^Red de Estudios Moleculares Avanzados, Clúster Biomimic®, Instituto de Ecología, A. C., Carretera Antigua a Coatepec No. 351, Colonia El Haya, 91070 Xalapa, VER, Mexico; ^6^Unidad de Servicios de Apoyo en Resolución Analítica, Universidad Veracruzana, 575 Xalapa, VER, Mexico

## Abstract

This study shows the seasonal effect on the antioxidant, antiproliferative, and antimicrobial activities of* L. glaucescens *Kunth (LG) leaves extracts. Their antioxidant activity was evaluated through the DPPH, FRAP, and ORAC assays. Their phenolic content (PC) was determined by means of the Folin-Ciocalteu method, and the main phenolic compounds were identified through a HPLC-DAD analysis. Antiproliferative activity was determined by MTT assay against HeLa, LS 180, M12.C3.F6, and ARPE cell lines. Antimicrobial potential was evaluated against* Staphylococcus aureus *and* Escherichia coli* using a microdilution method. All the LG extracts presented high antioxidant activity and PC, with quercitrin and epicatechin being the most abundant. Antioxidant activity and PC were affected by the season; particularly autumn (ALGE) and summer (SULGE) extracts exhibited the highest values (*p* < 0.05). All extracts presented moderate antiproliferative activity against the cell lines evaluated, HeLa being the most susceptible of them. However, ALGE and SULGE were the most active too. About antimicrobial activity, SULGE (MIC_90_ < 800 *μ*g/mL; MIC_50_ < 400 *μ*g/mL), and SLGE (MIC_50_ < 1000 *μ*g/mL) showed a moderate inhibitory effect against* S. aureus*. These findings provide new information about the seasonal effect on the PC and biological properties of LG extracts. Clearly, antioxidant activity was the most important with respect to the other two.

## 1. Introduction

Nowadays, diseases related to oxidative stress and to antimicrobial resistance are considered the main public health concern, leading to the highest mortality rates worldwide [1, 2]. Oxidative stress has been explained in terms of the overproduction of intracellular reactive oxygen species, which may produce damage to biomolecules such as DNA, RNA, lipids, and proteins [3]. Therefore, the cellular damage would eventually result in the development of chronic diseases like cancer, atherosclerosis, rheumatoid arthritis, diabetes, chronic inflammation, and cardiovascular ills, among others [4].

On the other hand, antimicrobial resistance is the result of antibiotic misuse, which conduces to stronger infections with complicated clinical treatments like respiratory tract infections, rhinosinusitis, otitis media, cystic fibrosis lung infection, dental caries, and chronic wounds, among others [5, 6]. These complications reduce the conventional antibiotics efficacy and length of the hospitalization stays and increase the medical treatment costs associated with the research and application of broad spectrum antibiotics [7]. Each year, around 2 million people are infected by antibiotic resistant bacteria in USA and thousands die due to infections with clinical complications [8]. In this context, natural agents emerge as a safe alternative to reduce the problem of the oxidative stress and antimicrobial diseases.

Plants are traditionally used in folk medicine to treat different illnesses and nearly 80% of worldwide population had used them with this purpose, especially for being a natural source easily available for the communities [9, 10]. Their positive health benefits are associated with the presence of chemical compounds derived from secondary metabolism, such as phenolic compounds, essential oils, terpenes, saponins, alkaloids, and polypeptides, which are used by plants as part of their defense mechanisms [11, 12]. In addition, these compounds had shown a broad spectrum of biological activities, demonstrating the potential of plants as alternative drugs [13, 14]. However, the content of bioactive compounds depends on biotic and abiotic factors such as the presence of microorganisms and competitor species around the plant, temperature, light intensity, UV radiation, humidity, water, minerals, and environmental contamination [15, 16]. These factors regulate the production of secondary metabolites and subsequently the potential use of medicinal plants [17]. In this sense, the study of the effect that the different seasons have on the chemical composition and biological properties of plants can contribute to their optimal use in the folk medicine [18, 19].

*Litsea glaucescens *Kunth is a native plant from Central America and Mexico, mainly distributed in the states of Chiapas Nayarit and Veracruz, where it is known as “laurel” [20]. Its leaves have been traditionally used as food seasoning, as well as remedy in folk medicine against central nervous system illness, depression, colic, pain, vomit, and diarrhea [21]. These activities are mainly related to the presence of different compounds such as terpenes and phenolic compounds [22, 23]. The goal of the present study was to evaluate the seasonal effect on the antioxidant, antimicrobial, and the antiproliferative activities of* L. glaucescens *Kunth leaves extracts, as well as on their content and profile of phenolic compounds, since to the best of our knowledge, this is the first effort to describe at this level the biological properties and chemical composition of “laurel,” commonly used as a remedy by the communities from the mountainous region of Veracruz, México.

## 2. Materials and Methods

### 2.1. Reagents

Folin-Ciocalteu's phenol reagent, sodium carbonate (Na_2_CO_3_), 1,1-diphenyl-2-picrylhydrazyl (DPPH), 6-hydroxy-2,5,7,8-tetramethylchroman-2-carboxylic acid (Trolox), 2,4,6-tri(2-pyridyl)-striazine (TPTZ), iron (III) chloride hexa-hydrate, sodium acetate trihydrate (C_2_H_3_NaO_2_·3H_2_O), hydrochloric acid, 2,2′-azobis(2-amidinopropane) dihydrochloride (AAPH), gentamicin, sodium chloride (NaCl), Dulbecco's Modified Eagle's Medium (DMEM) high glucose, dimethyl sulfoxide (DMSO), isopropyl alcohol, and 3-(4,5-dimethylthiazol-2-yl)-2,5-diphenyltetrazolium bromide (MTT), as well as authentic standards of epicatechin, quercitrin, gallic acid, chlorogenic acid, caffeic acid,* trans*-cinnamic acid, scopoletin, hesperidin, rosmarinic acid, myricetin, genistein, luteolin, and apigenin were purchased from Sigma-Aldrich (St. Louis, MO, USA). Standards of naringenin, hesperetin, chrysin, galangin, and acacetin were purchased from INDOFINE Chemical Co., Inc. (Hillsborough, NJ, USA). Mueller-Hinton broth (MHB) and Mueller-Hinton agar (MHA) were obtained from Becton Dickinson (Cockeysville, MD, USA). HPLC-grade water (18 mΩ) was performed by a Milli-Q50 purified system (Millipore Corp., Bedford, MA, USA).

### 2.2. Plant Material and Preparation of Extracts

*L. glaucescens* leaves were collected during autumn (November 2015), winter (February 2016), spring (May 2016), and summer (September 2016) in Xico, Veracruz, México.* L. glaucescens* leaves were identified in the Herbarium of Instituto de Investigaciones Biológicas of Universidad Veracruzana, México. Collected leaves were washed and dried. Dried leaves were extracted with methanol (96%) during 4 days with occasional stirring (2-3 times per day). The extracts were filtered using filter paper (Whitman grade number 4) and the solvent was evaporated to dryness under reduced pressure at 40°C in a rotary evaporator. The obtained extracts were stored at −20°C and identified as* L. glaucescens* autumn, winter, spring, and summer extracts (ALGE, WLGE, SLGE, and SULGE, resp.).

### 2.3. Total Phenolic Content

Total phenolic concentration was determined with Folin-Ciocalteu reagent, according to the method described by Velazquez et al. [24]. Briefly, 10*μ*L of extracts (1 mg/mL) was mixed with 80*μ*L of distilled water, 40*μ*L of Folin-Ciocalteu reagent 0.25 N, 60*μ*L sodium carbonate (5% in distilled water), and 80*μ*L of distilled water. The mixtures were incubated at room temperature (1 h). The absorbance of the samples was measured at 750 nm on a Fluostar Omega microplate reader (BMG Labtech, Ortenberg, Germany), and the results were expressed as milligrams of gallic acid equivalent (GAE)/gram of dry weight (d. w.).

### 2.4. HPLC-DAD Analysis

Analytical HPLC-DAD analysis was carried out on an Agilent 1220 Infinity DAD LC (Waldbronn, Germany) equipped with a Zorbax SB-C18 column (250 × 4.6 mm, Ø 3.5*μ*m, Agilent, USA). The mobile phase consisted of 5% formic acid in water (solvent A) and methanol (solvent B). The elution was accomplished with a solvent flow rate of 1 mL/min, using a gradient program as follows: 5% B (0–5 min), 10% B (5–10 min), 15% B (10–18 min), 25% B (18–28 min), 30% B (28–40 min), 40% B (40–45 min), 45% B (45–55 min), 60% B (55–60 min), 80% B (60–65 min), 100% B (65–76 min), and 30% B (76–86 min). Flavonoids were monitored at 280 and 340 nm. Identification of phenolic compounds was carried out by comparison of the retention times and spectra with those of authentic standards. Quantification of both compounds was performed through calibration curves. Results were expressed as mg of each compound/100 mg of d.w.

### 2.5. DPPH Assay

Free-radical scavenging activity was measured following the modified method reported by Usia et al. [25].* L. glaucescens* extracts (100*μ*L) were mixed with a 300*μ*M DPPH solution (100*μ*L). Samples were kept in the dark for 30 min. Afterward, absorbance at 517 nm was measured on a microplate reader (Fluostar Omega microplate reader, BMG Labtech Ortenberg, Germany).* L. glaucescens* extracts were tested at different concentrations (0 to 100*μ*g/mL). Results were expressed as *µ*M of trolox equivalents (TE)/g of d.w. and IC_50_. IC_50_ values were calculated throughout linear regression analysis using Microsoft Excel software.

### 2.6. FRAP Assay

Ferric reducing ability was evaluated according to the methodology described by Benzie and Strain [26]. Working FRAP reagent was elaborated reacting 10 volumes of 300 mM acetate buffer (pH 3.6), 1 volume of 40 mM TPTZ (dissolved in 40 mM HCl), and 1 volume of 20 mM ferric chloride (dissolved in water). Subsequently, 280*μ*L of FRAP reagent was mixed with 20*μ*L (0.5 mg/mL) of* L. glaucescens* extracts, and the absorbance was read at 630 nm at a microplate reader (Fluostar Omega microplate reader, BMG Labtech Ortenberg, Germany) after 30 min of storage in the dark. Results were reported as *µ*M of Fe(II)/g of d.w.

### 2.7. ORAC Assay

Oxygen radical absorbance capacity assay was carried out using a modified method described by Ou et al. [27]. AAPH reagent was used as peroxyl radical generator and fluorescein as the fluorescent indicator. Reaction mixture contained 150*μ*L of fluorescein (10 nM), 25*μ*L of phosphate buffer (75 mM, pH 7.4) as blank, and 25*μ*L (50*μ*g/mL) of extracts. Reaction was started by the addition of AAPH (240 mM). Samples were preincubated at 37°C (15 min) and the fluorescence was monitored every 90 s for 1.5 h at 485–520 nm on a microplate reader (Fluostar Omega microplate reader, BMG Labtech Ortenberg, Germany). Results were expressed as*μ*M TE/g d.w.

### 2.8. Bacterial Strains and Growth Conditions

*Escherichia coli* ATCC 25922 and* Staphylococcus aureus* ATCC 25923 were employed in the experiments. These strains were maintained at −70°C in cryovials containing glycerol (10%) broth and subculture in Mueller-Hinton broth, at 37°C during 24 h before testing.

### 2.9. Antibacterial Assay

Antibacterial activity of extracts was evaluated by the modified microdilution broth method [24]. Briefly, after overnight growth at 37°C in Mueller-Hinton agar, 15*μ*L (1.5 × 10^6^ CFU) of a suspension of a logarithmic phase bacterial culture [10^8^ CFU ml^−1^, the turbidity of this bacterial suspension matching the turbidity of a 0.5 McFarland standard] was inoculated into each well of a flat 96-well microplate (Costar, Corning, NY, USA), containing 200*μ*L of different concentrations (100–1000*μ*g/mL) of* L. glaucescens*. The extracts were dissolved previously in DMSO and subsequently diluted in sterile MHB. The percentage of DMSO did not exceed 2% (v/v) of the total volume per well (215*μ*L). Gentamicin (12*μ*g/mL) was used as positive control of bacterial growth inhibition. Plates were incubated for 48 h at 37°C and read later at 620 nm, on a microplate reader (Multiskan EX, ThermoLab System), at 6, 12, 24, and 48 h. The minimal inhibitory concentration was defined as the lowest extracts concentration that inhibited at least 50% (MIC_50_) or 90% (MIC_90_) of the bacterial growth after incubation (37°C × 24 h). MICs values were calculated from the Optical Density (OD_620 nm_) data using the following equations:

(1) MIC50:  OD620 nm  untreated bacteria−OD620 nm  test concentrationOD620 nm  untreated bacteria×100≥50%MIC90:  OD620 nm  untreated bacteria−OD620 nm  test concentrationOD620 nm  untreated bacteria×100≥90%.

### 2.10. Cell Lines

Cell lines LS 180 (human colonic adenocarcinoma), HeLa (human cervix carcinoma), and ARPE-19 (human retinal pigmented epithelium) were obtained from the American Type Culture Collection (ATCC; Rockville, MD, USA). The cell line M12.C3.F6 (murine B-cell lymphoma) was provided by Dr. Emil R. Unanue (Department of Pathology and Immunology, Washington University in St. Louis, MO, USA). Cells were cultured in DMEM supplemented with 5% FBS (Sigma, St. Louis, MO, USA).

### 2.11. Antiproliferative Assay

Cell proliferation was evaluated through the MTT assay [28] modified by Hernandez et al. [29]. Briefly, 50*μ*L (1 × 10^4^ cells) was placed in each well of a flat 96-well plate and incubated for 24 h (37°C, 5% of CO_2_ atmosphere). Then, 50*μ*L of medium containing different concentrations of extracts was added and the cell cultures were incubated for 48 h. Extracts were previously dissolved in DMSO. DMSO did not exceed 0.5% of the total volume per well (preliminary studies showed that DMSO at this concentration does not cause damage of cell). Caffeic acid phenethyl ester (CAPE) was used as a positive control in the antiproliferative assay. In the last 4 h of the LS 180, HeLa, and ARPE cell line cultures, each well was washed with PBS and refilled with new fresh culture medium. Subsequently, 10*μ*L of a MTT solution (5 mg/mL) was added to each well (in the case of the M12.C3.F6 cell line culture, only MTT solution (5 mg/mL) was added). Metabolically active cells reduced tetrazolium salt to colored formazan crystals, which were dissolved with acidic isopropyl alcohol. Microplates were read at 570 and 650 nm (Multiskan EX, ThermoLab System). Results were expressed as IC_50_ values (IC_50_ is defined as the required concentration to inhibit 50% of the cell proliferation). Graphic of living cells (%) versus extracts concentrations was traced. IC_50_ values were calculated using nonlinear regression analysis in Microsoft Excel software.

### 2.12. Statistical Analysis

Data analysis was performed using the NCCS, 2007, statistical software. One-way ANOVA was used, and mean comparisons were performed using the Tukey-Kramer test. Significance level in Type I error was *p* ≤ 0.05. Pearson correlation between phenolic content and DPPH, FRAP, and ORAC values were estimated too. 

## 3. Results and Discussion

### 3.1. Phenolic Compounds

**Figure 1:**
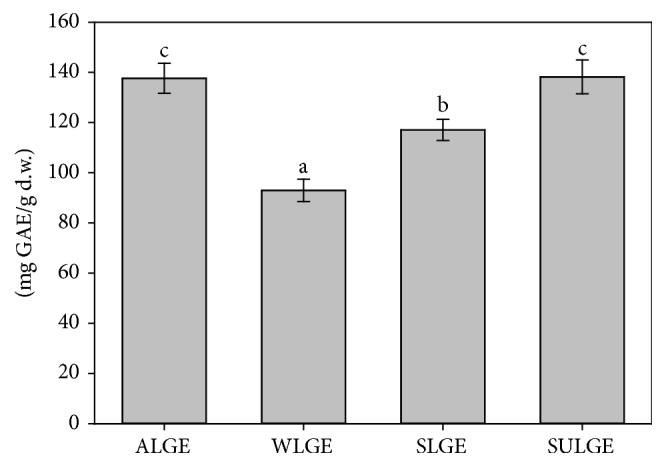
Phenolic content of* L. glaucescens* extracts. ^a–c^Bars with different superscript indicate statistical differences (*p* < 0.05) (ALGE: autumn* L. glaucescens *extract; WLGE: winter* L. glaucescens* extract; SLGE: spring* L. glaucescens* extract; SULGE: summer* L. glaucescens* extract).

The phenolic content of the* L. glaucescens* extracts ranged from 92.9 ± 4.4 to 138.2 ± 6.7 mg GAE/g d.w. The highest concentrations (*p* < 0.05) were found in ALGE and SULGE followed by SLGE and WLGE (Figure 1). These data agree with those reported by Iqbal and Bhanger [30], Brahmi et al. [31], and Sivaci and Duman [32], who evaluated the seasonal effect of phenolic content of moringa, olive, and almond leaves extracts, respectively. In the three studies, they found that autumn extracts presented the highest phenolic concentrations, in comparison with the samples of the other seasons.

**Figure 2:**
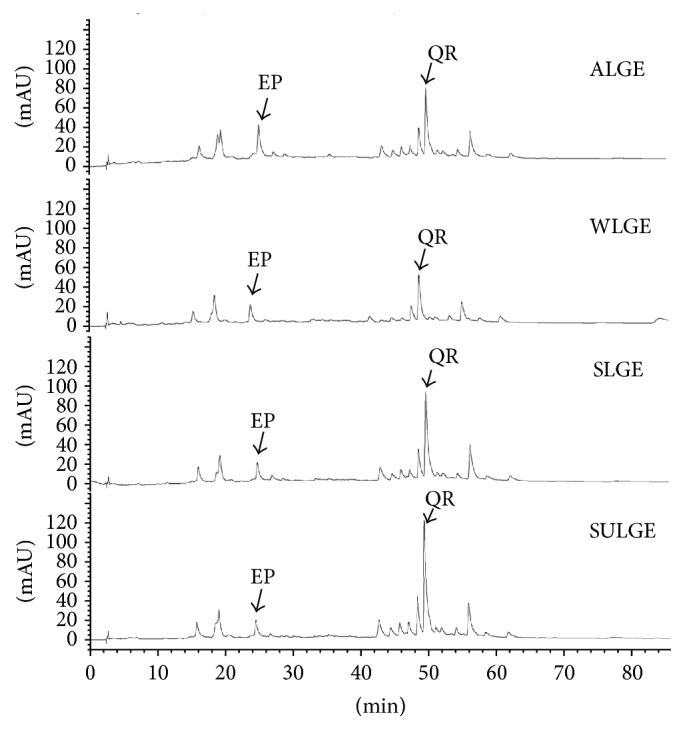
HPLC chromatogram of* L. glaucescens* extracts (recorded at 280 nm) (EP: epicatechin; QR: quercitrin) (ALGE: autumn* L. glaucescens *extract; WLGE: winter* L. glaucescens* extract; SLGE: spring* L. glaucescens* extract; SULGE: summer* L. glaucescens* extract).

To identify the main phenolic compounds of the extracts of* L. glaucescens, *a HPLC-DAD analysis was performed. The chromatographic profiles of the four seasonal extracts are shown in Figure 2. As can be observed, the evident difference among them is the height of the chromatographic peaks (related to the concentration of the phenolics). Comparison of the retention times and spectra with those from a set of commercial standards allowed us to identify two of the main phenolic compounds present in* L. glaucescens* extracts: epicatechin and quercitrin, which present quantitative variation in* L. glaucescens* throughout the year (Figure 2). Quercitrin was the most abundant phenolic compound in the four extracts, and to the best of our knowledge, this is the first time that it is reported as a component of* L. glaucescens*. SULGE presented the highest amount (*p* < 0.05) of quercitrin, followed by SLGE, ALGE, and WLGE (Table 1). Epicatechin, the second-major flavonoid found in the extracts, has been reported before by Gamboa-Gómez et al. [33] as a secondary metabolite of* L. glaucescens*. In this work, the ALGE extract presented the highest (*p* < 0.05) epicatechin content, while a similar amount (*p* > 0.05) of this compound was observed in the other extracts.

Phenolic composition of plants is mainly affected by biotic and abiotic factors. In normal conditions, abiotic factors such as thermal stress play an important role in the biosynthesis of phenolic compounds in plants, because they induce the phenylalanine ammonia-lyase (PAL) activation, which is the main enzyme involved in the biosynthesis of phenylpropanoid [34, 35]. In addition, the increase in the enzymatic activity of PAL is related to an adaptation of the plant to stress [36]. Therefore, it is possible to hypothesize that* L. glaucescens* was subjected to a higher thermal stress during summer and autumn, compared with spring and winter, resulting in an increase of phenolic compounds during these seasons. On the other hand, phenolic compounds are associated with a wide range of biological activities. To contribute to the biological characterization of this plant, we evaluated its potential as antioxidant, antimicrobial, and antiproliferative agent.

### 3.2. Antioxidant Activity

Different assays are available and have been used to evaluate the antioxidant activity of plant extracts. Most of them are based on scavenging specific radicals such as DPPH and peroxyl radicals or metal reducing potential such as the FRAP assay. In the present study, we evaluated the antioxidant activity of* L. glaucescens* extracts throughout three chemicals assays DPPH, FRAP, and ORAC (Table 2). Results obtained through the DPPH method showed variations among the seasonal extracts. From the IC_50_ values it is possible to observe that ALGE and SULGE were the most active samples (*p* < 0.05) against the DPPH radical, in comparison with WLGE and SLGE. In addition, based on Blois [37] and Fidrianny et al. [38] classifications, the antioxidant capacity of the four extracts must be categorized as very strong, since all of them had IC_50_ values lower than 50*μ*g/mL. These results agree with previous studies related to plants from* Litsea *genus such as* Litsea glutinosa, Litsea floribunda*, and* Litsea japonica, *where IC_50_ values ranged from 9.68 to 669.2*μ*g/mL [39–41].

On the other hand, ferric reducing power of the* L. glaucescens* extracts was evaluated through their ability to reduce the ferric complex Fe3+-tripyridyltriazine to Fe2+-tripyridyltriazine. The corresponding values are shown in Table 2; and as can be observed there are significant differences among them (*p* < 0.05). Particularly, ALGE and SULGE exhibited the stronger power, whereas WLGE had the lowest activity. These values are higher than those reported before for other* Litsea *species (1.4–638*μ*M Fe(II)/g of d.w.) [40, 42, 43]. In addition, according to the classification of medicinal plants performed by Wong et al. [44], the* L. glaucescens* extracts had an extremely high ferric reduction power, since the obtained values were higher than 500*μ*M Fe(II)/g of d.w.

The capacity of* L. glaucescens *extracts to scavenge the AAPH-derived peroxyl radical was evaluated through the ORAC assay and the results are shown in Table 2; similar values were obtained for SULGE, SLGE, and ALGE, while the lowest one was registered for WLGE. Although all the extracts had strong antioxidant activity, the results presented here demonstrated that during the winter the capacity of* L. glaucescens *to react with peroxyl radical decreased.

The three types of tests performed in this study provided evidences about the high ability of the four extracts to transfer electron and hydrogen atoms to stabilize free radicals and reduce metals, related to their strong antioxidant activity. In addition, the significant effect that the seasons had on the antioxidant capacity of* L. glaucescens *extracts is notorious. Particularly, ALGE and SULGE showed a higher activity, with respect to SLGE and WLGE. Additionally, the four extracts exhibited an interesting ability to act as preventive and chain-breaking antioxidants with activity against biological and synthetic radicals. These facts suggest that they have the potential to stabilize biological radicals and to inhibit the generation of reactive oxygen species, which could contribute to reducing the oxidative stress caused by them and therefore to avoiding the DNA damage.

**Figure 3:**
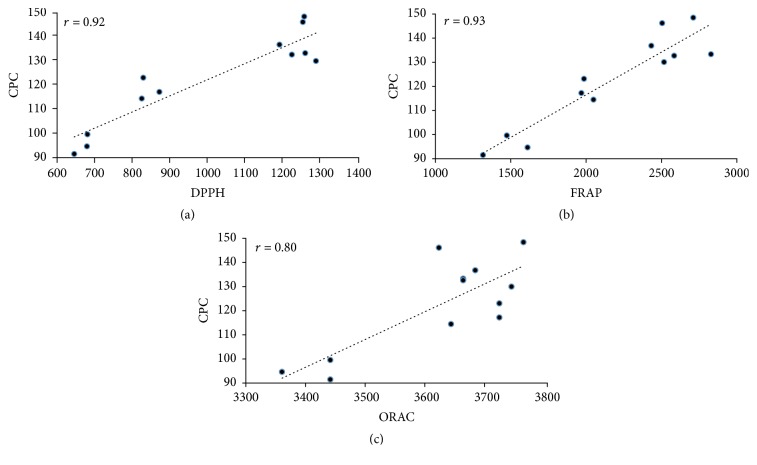
Plots of the data obtained through the DPPH, FRAP, and ORAC assays against the concentration of phenolic compound (CPC) in the four* L. glaucescens* extracts. The correlation coefficients (*r*) are shown in each graph.

**Figure 4:**
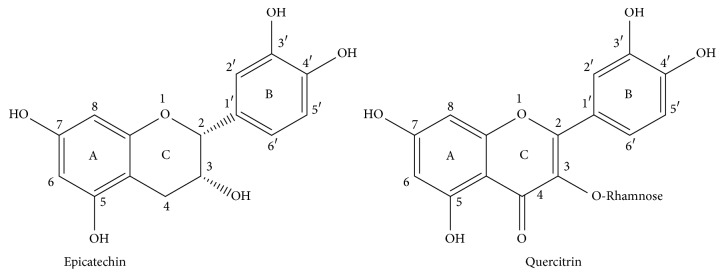
Structure of identified phenolic compounds in* L. glaucescens* extracts.

It is well known that the antioxidant activity of natural products is strongly related to the content of phenolic compounds that they have, and the results obtained in this work agree with that fact. In order to demonstrate the correlation between both parameters, a series of plots of the three data pieces obtained through the DPPH, FRAP, and ORAC assays for each* L. glaucescens* extract (the samples were evaluated in triplicate) against the corresponding averaged concentration of phenolic compound (CPC) were performed (Figure 3). The regression coefficients (*r*) of the linear correlations for each series are presented in Figure 3. As can be observed, positive slopes were obtained in all the cases. On the other hand, the lowest regression coefficient was obtained for the correlation between the data of the ORAC test versus CPC; however the value is still into an acceptable range. As we hypothesized, it seems that phenolic compounds were the main compounds responsible for the antioxidant activity of the four* L. glaucescens* extracts. Although the evaluated samples have other phenolic constituents not identified in this work, it is possible that epicatechin and quercitrin could play an important role in the high antioxidant activity of ALGE and SULGE, since different studies have demonstrated that both phenolics are considered among the most antioxidant phenolic compounds [45, 46] and that capacity has been attributed to the catechol and chromane moieties that they have (Figure 4). Particularly, the presence of hydroxyl groups in 3′- and 4′-position of ring B, hydroxyl group 3 of ring C, and double bond between C2 and C3 enhance the antioxidant activity of these phenolic compounds, since they can transfer electrons and protons to stabilize free radicals or to reduce and chelate metals. These structural features confer to both compounds a greater stability, compared with those that lack them [13, 47]. In addition, these facts determine also the redox potential and therefore the antioxidant activity of phenolic compounds [48].

### 3.3. Antiproliferative Activity

The results of the antiproliferative activity evaluation of* L. glaucescens* extracts against HeLa, LS 180, M12.C3.F6, and ARPE cells are shown in Table 3. Although all the extracts inhibited the proliferation of human and murine cells lines, their effect was moderate. As can be observed in Table 3, HeLa was the more sensitive cell line to the* L. glaucescens* extracts, particularly to the SULGE and ALGE ones, which exhibited the highest activity (*p* < 0.05) against its proliferation. Regarding LS 180, ALGE and SULGE showed the stronger activity too (*p* < 0.05), whereas in the case of the cancerous murine cell line (M12.C3.F6), the four extracts had a similar antiproliferative effect (*p* > 0.05). In addition, ALGE showed the lower IC_50_ value (*p* < 0.05) to inhibit the proliferation of the noncancerous ARPE cell line; however much higher concentrations of SLGE, SULGE, and WLGE were required. Even more, these last three values are the highest of Table 3 and constitute an evidence of the selectiveness of the* L. glaucescens* extracts to inhibit the proliferation of cancerous cell lines with respect to those noncancerous ones. These findings are consistent with previous studies from* Litsea* plants. For example, in a study performed by Herrera-Carrera et al. [49], it was demonstrated that a herbal infusion obtained from* L. glaucescens* was able to inhibit the proliferation of human colon cancer cell line (HT-29). In the same way, Ndi et al. [50] observed inhibition on HT-29 (IC_50_ = 37.9*μ*g/mL) and melanoma (SK-MEL-28) (IC_50_ > 100*μ*g/mL) cells treated with of* L. glutinosa* extract. Subarnas et al. [51] evaluated the antiproliferative activity of* L. mappaceae* extracts against human breast cancer (MCF-7) and reported that 200*μ*g/mL of plant extract was required to inhibit the 50% of cell proliferation. Although in all the cases the positive control CAPE was several times more active than the* L. glaucescens* extracts (see Table 3), it should be kept in mind that the purity of the CAPE used in the assays (above 95%) is much higher than those of the active compounds present in the extracts and this fact could contribute to the differences observed in the IC_50_ values.

The results reported in this work are an evidence of the antiproliferative effect of* L. glaucescens* against cancerous cell lines, in comparison with those noncancerous ones. In addition, the influence of seasons on its antiproliferative activity was significatively different in three of the four cell lines studied here. However, in some cases the registered differences were still modest. ALGE and SULGE were the more active extracts, which could be related to their high content of phenolic compounds. Nevertheless, the structural features of these phenolics are important too such as those described above as enhancer of the antioxidant activity of the* L. mappaceae* extracts [52]. In this sense, Kinjo et al. [53] and Nagarajan et al. [54] proposed that epicatechins, one of the most abundant phenolics of the four extracts reported here, possess potent antiproliferative activity against cancerous cells lines, which was related to an arrest of the cell cycle in the G2 phase. On the other hand, previous studies have reported that phenolic compounds exhibit different mode of actions against cancerous cell lines, for example, the induction of apoptosis, the cell cycle arrest, and the prevention of carcinogen metabolic activation with the subsequent cell death [55, 56].

### 3.4. Antimicrobial Activity

**Figure 5:**
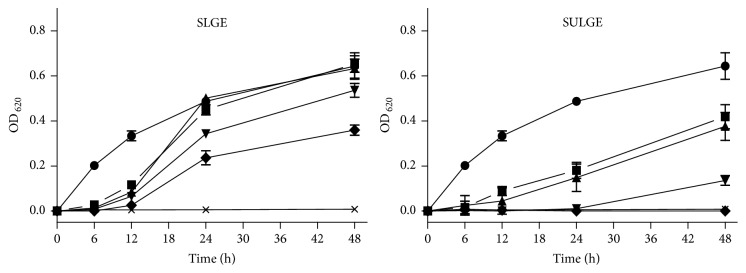
Antibacterial activity of* L. glaucescens* extracts against* Staphylococcus aureus* (SLGE: spring* L. glaucescens* extract; SULGE: summer* L. glaucescens* extract). Bacterial cell cultures were treated with different concentrations of* L. glaucescens* extracts during 48 h. ◆: 1000 *μ*g/mL; ▼: 800 *μ*g/mL; ▲: 600 *μ*g/mL; ■: 400 *μ*g/mL; ●: 0 *μ*g/mL;** ×**: gentamicin. Control bacterial cultures were incubated with DMSO (0.8–2%). Gentamicin (12 *μ*g/mL) was used as positive control. All values represent mean ± standard deviation.

Results of antibacterial activity of* L. glaucescens* extracts are summarized in Table 4 and Figure 5. As can be observed from Table 4, SULGE and SLGE showed the strongest activity against* S. aureus*, since the MIC_50_ values obtained for both are below the maximum concentration evaluated (1000*μ*g/mL), which constitutes an evidence of the important seasonal effect on the biological properties of* L. glaucescens*. In contrast, no antimicrobial activity of any of the four extracts was observed against* E. coli*. The fact that* S. aureus* (Gram-positive) was less resistant than* E. coli* (Gram-negative) to* L. glaucescens* could be attributed to the cell structure and composition of both types of microorganisms. In this regard, it has been proposed by other authors that the outer membrane of Gram-negative bacteria, constituted by phospholipid and lipopolysaccharides, provides resistance to antimicrobial treatments [57]. On the other hand, the porins in outer membrane could regulate too the penetration of hydrophilic substances and reduce the fluidity of the lipopolysaccharides layer, decreasing the rate of transmembrane diffusion [58]. According to those studies, the Gram-positive bacteria would have lesser resistance to antimicrobial treatments.

In addition, the dose-depend relationships of the active extracts (SULGE and SLGE) against* S. aureus* were explored, and the corresponding plots are shown in Figure 5. As can be observed, there is a clear effect of the concentration of both on their antimicrobial activity. SULGE, the most potent extract, was able to inhibit 100% of bacteria growth at the highest tested concentration (1000*μ*g/mL), 98% at 800*μ*g/mL, 70% at 600*μ*g/mL, and up to 63% at 400*μ*g/mL. In addition, higher concentrations, such as 1000 and 800*μ*g/mL, had a similar activity with respect to gentamicin (>98% inhibition). Although in a minor proportion than SULGE, SLGE also exhibited antimicrobial effect depending on concentration against* S. aureus*. As can be observed from Figure 5, a concentration of 1000*μ*g/mL inhibited 51% of bacteria growth, while other concentrations provoked an inhibition lower than 50%. Phytochemicals are classified as antimicrobials based on susceptibility test achieving inhibitory concentrations in the range of 100 to 1000*μ*g/mL. Thus, extracts with MIC values equal to or lower than 100*μ*g/mL are considered effective antimicrobial agents, while those with MIC values between 100 and 500*μ*g/mL and between 500 and 1000*μ*g/mL are known as moderate and low antimicrobial agents, respectively. Extracts with MIC values above 1000*μ*g/mL do not have antimicrobial activity [59, 60]. In this sense, SULGE and SLGE showed moderate to low antimicrobial activity against* S. aureus*, while the other extracts were considered inactive against this bacterium.

On the other hand, the antibacterial activity of* L. glaucescens* extracts evaluated in the present research is consistent with previous works about the* Litsea* genus. For example, in a study performed by Ali-Ahmmad et al. [61]* L. monopetala* extracts presented inhibitory effect against* S. aureus* and* E. coli* at concentrations of 62.5 and 250*μ*g/mL, respectively. Similarly, Areekul et al. [62] evaluated the antimicrobial activity of a* L. glutinosa* extract at 6.97% (w/v) against* S. aureus* and* E. coli*. Although lower in comparison to the effect of chloramphenicol, which was used as positive control, the* L. glutinosa* extract showed activity against* S. aureus* (21.34 mm and 8.78 mm of inhibition, resp.). Nevertheless, it was not able to inhibit the growth of* E. coli*. In addition, Pradeepa et al. [63] reported a behavior close to the results reported here, since they observed that the* L. glutinosa* extracts presented greater antimicrobial activity against* S. aureus* (MIC = 2.5 mg/mL) compared with* E. coli* (MIC = 5 mg/mL).

Antimicrobial effect of active* L. glaucescens extracts* could also be related to the high phenolic compounds content. In this regard, Borges et al. [64] and Andrade et al. [65] demonstrated that phenolic compounds induced alteration of membrane properties, producing changes in the hydrophobicity, surface charge, and membrane integrity with the subsequent leakage of essential intracellular constituents of Gram-positive and Gram-negative bacteria. On the other hand, Cushnie and Lamb [66, 67] have concluded that phenolic compounds have different mechanism of action as antimicrobial, such as inhibitors of nucleic acid synthesis, of the energy metabolism, or of the cytoplasmic membrane function of the microorganisms.

## 4. Conclusions

This research provides information about seasonal effect on the concentration of phenolic compounds (particularly epicatechin and quercitrin) present in* L. glaucescens* extracts, as well as on a series of biological activities that they have. From the results reported here, the antioxidant activity was the strongest one and clearly dependent on the season. Therefore, the* L. glaucescens* extracts could be a promising alternative to avoid oxidative stress. On the other hand, antiproliferative and antimicrobial activities were moderate. The first of them increased slightly during the autumn and summer, which is related to the major concentration of phenolic compounds produced by the plant as response to the environmental conditions. In addition,* L. glaucescens* extracts collected during summer and spring showed moderate antimicrobial activity against* S. aureus*, which may be related to the high quercitrin concentration produced by the plant during those seasons. Despite the interesting properties that this Mexican plant has, subsequent studies are required to support its effectiveness and safety doses for human applications.

## Figures and Tables

**Table 1:** Concentration of major phenolic compounds identified in *L. glaucescens* extracts.

Compound	*L. glaucescens* extracts (mg/100 mg d.w.)			
	ALGE	WLGE	SLGE	SULGE
Epicatechin	1.56 ± 0.19^b^	0.88 ± 0.009^a^	0.73 ± 0.02^a^	0.68 ± 0.01^a^
Quercitrin	2.11 ± 0.05^b^	1.39 ± 0.17^a^	3.01 ± 0.16^c^	3.89 ± 0.32^d^

^a–d^Means with different superscript within the same row indicate statistical differences (*p* < 0.05). All values represent mean ± standard deviation (ALGE: autumn *L. glaucescens *extract; WLGE: winter *L. glaucescens* extract; SLGE: spring *L. glaucescens* extract; SULGE: summer *L. glaucescens* extract).

**Table 2:** Antioxidant activity of *L. glaucescens* extracts.

Extract	Antioxidant assay			
	DPPH (*µ*M TE/g of d.w.)	DPPH (IC_50_, *µ*g/mL)	FRAP (*µ*M Fe (II)/g of d.w.)	ORAC (*µ*M TE/g of d.w.)
ALGE	1264.5 ± 18.5^c^	14.7 ± 0.07^c^	2614.3 ± 183.1^c^	3673.3 ± 61.1^b^
WLGE	668.1 ± 19.9^a^	27.2 ± 0.8^a^	1466.4 ± 147.6^a^	3413.3 ± 46.1^a^
SLGE	841.1 ± 25.9^b^	24.3 ± 0.9^b^	1999.7 ± 42.4^b^	3693.3 ± 46.1^b^
SULGE	1221.9 ± 32.6^c^	15.2 ± 0.3^c^	2573.4 ± 138.9^c^	3700.3 ± 52.9^b^

^a–c^Means with different superscript within the same column indicate statistical differences (*p* < 0.05). All values represent mean ± standard deviation (ALGE: autumn *L. glaucescens *extract; WLGE: winter *L. glaucescens* extract; SLGE: spring *L. glaucescens* extract; SULGE: summer *L. glaucescens* extract).

**Table 3:** Antiproliferative activity of *L. glaucescens* extracts.

Cell line	*L. glaucescens* extracts IC_50_ (*µ*g/mL)				
	ALGE	WLGE	SLGE	SULGE	CAPE (*µ*g/mL/*µ*M)
HeLa	48.7 ± 1.8^b^	53.9 ± 2.6^bc^	59 ± 7.8^c^	45.8 ± 1.6^b^	9.7 ± 0.07^a^/34.1 ± 0.2
LS 180	53.1 ± 1.2^a^	85.2 ± 3.5^c^	67.5 ± 3.9^b^	55.6 ± 1.5^a^	17.8 ± 0.2^a^/62.6 ± 0.7
M12.C3.F6.	71.9 ± 6.2^b^	70.6 ± 2.1^b^	73.2 ± 2.5^b^	68.1 ± 1.3^b^	0.58 ± 0.04^a^/2.04 ± 0.1
ARPE	62.1 ± 3.6^b^	166.1 ± 4.9^d^	101.9 ± 5.6^c^	102.2 ± 1.9^c^	10.2 ± 0.18^a^/35.9 ± 0.6

^a–d^Means with different superscript within the same row indicate statistical differences (*p* < 0.05). All values represent mean ± standard deviation (ALGE: autumn *L. glaucescens *extract; WLGE: winter *L. glaucescens* extract; SLGE: spring *L. glaucescens* extract; SULGE: summer *L. glaucescens* extract).

**Table 4:** Growth-inhibitory activity of *L. glaucescens* extracts against *S. aureus* and *E. coli*.

Strain	*L. glaucescens* extracts (*µ*g/mL)							
	ALGE		WLGE		SLGE		SULGE	
	MIC_50_	MIC_90_	MIC_50_	MIC_90_	MIC_50_	MIC_90_	MIC_50_	MIC_90_
*S. aureus*	>1000	>1000	>1000	>1000	<1000	>1000	<400	<800
*E. coli*	>1000	>1000	>1000	>1000	>1000	>1000	>1000	>1000
